# Using interpretable machine learning model to predict lymph node metastasis in patients with localized prostate cancer

**DOI:** 10.3389/fonc.2026.1804019

**Published:** 2026-07-22

**Authors:** Tianwei Zhang, Pengfeng Gong, Shang Xu, Ruize Qin, Zhilong Liu, Jingxian Wang, Xinning Wang, Wei Jiao

**Affiliations:** 1 Department of Urology, The Affiliated Hospital of Qingdao University, Qingdao, China; 2 Department of Urology, The Third Affiliated Hospital of Soochow University, Changzhou, China

**Keywords:** lymph node metastasis, machine learning, prediction model, prostate cancer, SHAP value

## Abstract

**Background:**

This study aimed to develop and externally validate machine learning (ML) models for predicting lymph node metastasis (LNM) in patients with localized prostate cancer (PCa) using routinely available preoperative variables.

**Methods:**

Patients with localized PCa who underwent radical prostatectomy with extended pelvic lymph node dissection were retrospectively recruited from two institutions. The primary cohort comprised patients from the Affiliated Hospital of Qingdao University, while patients from the Third Affiliated Hospital of Soochow University were included as an external validation cohort. The primary cohort was randomly allocated into training (70%) and internal validation (30%) cohorts. Five ML algorithms were used to develop prediction models. Model performance was evaluated using the area under the receiver operating characteristic curve (AUC), and pairwise differences in AUC were assessed using DeLong’s test. The optimal model was compared with the established Briganti and Memorial Sloan Kettering Cancer Center (MSKCC) nomograms. SHapley Additive exPlanations (SHAP) were utilized to enhance model interpretability and quantify the relative contribution of individual features.

**Results:**

A total of 632 patients were included in the primary cohort, and 395 patients were included in the external validation cohort. In the internal validation cohort, the LightGBM model significantly outperformed the other candidate ML algorithms (DeLong test, p < 0.05) and achieved an AUC of 0.8613 (95% CI: 0.7976-0.9251). In the external validation cohort, LightGBM maintained the highest AUC, reaching 0.8148 (95% CI: 0.7477-0.8819). Furthermore, the LightGBM model showed predictive performance comparable to that of the established Briganti and MSKCC nomograms. SHAP analysis demonstrated that total prostate-specific antigen, body mass index, biopsy gleason grade group, neutrophil-to-lymphocyte ratio, systematic prostate biopsy positive rate, neutrophil percentage-to-albumin ratio, clinical T stage, and platelet-to-lymphocyte ratio were the key contributors to the model’s predictive performance.

**Conclusions:**

The LightGBM model incorporating eight key variables achieved competitive performance and showed predictive ability comparable to that of established Briganti and MSKCC nomograms. This model may serve as a useful preoperative decision-support tool, although prospective validation in larger and more diverse populations is still required before routine clinical implementation.

## 1 Introduction

As the most common malignancy of the urinary tract, prostate cancer (PCa) remains a significant cause of cancer-related mortality in men worldwide (1, 2). Radical prostatectomy (RP), often combined with extended pelvic lymph node dissection (ePLND), is widely used in the management of intermediate-risk and high-risk PCa, with ePLND serving as the reference standard for nodal staging (3). Lymph node metastasis (LNM) has been consistently identified as a key determinant of prognosis in localized PCa, correlating strongly with biochemical recurrence, distant metastasis, and cancer-specific mortality (4, 5). However, accurate preoperative assessment of lymph node status remains challenging in routine clinical practice. More than 80% of patients undergoing ePLND are ultimately found to have no nodal metastases, meaning that the majority are exposed to unnecessary surgical trauma and complication risks without any oncologic benefit (6). In addition, ePLND is associated with substantial morbidity, including lymphocele formation, thromboembolic events, and vascular and nerve injuries (7, 8). Conversely, failure to identify patients with occult nodal disease may delay appropriate treatment intensification and increase the risk of disease progression (9). Together, these limitations highlight an urgent need for more accurate preoperative risk stratification tools to guide patient selection for lymph node dissection and optimize individualized treatment strategies.

To guide patient selection for ePLND, several prediction tools have been developed, most notably the Briganti nomogram and the Memorial Sloan Kettering Cancer Center (MSKCC) nomogram (10, 11). However, these tools are largely based on conventional regression frameworks, which rely on assumptions of linearity and additivity among predictors. Such assumptions limit their ability to capture the complex, nonlinear interactions that characterize tumor biology and metastatic progression, resulting in suboptimal predictive performance. Imaging modalities, including computed tomography (CT), magnetic resonance imaging (MRI), multiparametric MRI (mpMRI), and prostate-specific membrane antigen positron emission tomography (PSMA PET/CT), have been increasingly used for nodal staging (12, 13). While these techniques offer valuable anatomic and functional information, their sensitivity for detecting microscopic lymph node metastases remains limited, particularly for nodes smaller than 5 mm. Consequently, imaging alone is insufficient for reliable exclusion of nodal disease.

Recent studies have increasingly highlighted the role of machine learning (ML) in PCa management. Yang et al. proposed a clinical machine-learning nomogram based on peripheral lymphocyte subsets for PCa risk stratification, suggesting that immune-related features may improve individualized assessment (14). Cao et al. subsequently reported a nonlinear transformation stacking learning strategy for PCa risk stratification, further supporting the value of nonlinear and ensemble-based approaches in this setting (15). In parallel, Han et al. demonstrated the growing promise of ML-assisted molecular testing for noninvasive PCa detection (16). More recently, Özlü et al. developed ML models for predicting PCa in biopsy candidates by integrating prostate-specific antigen, magnetic resonance imaging, and hematological parameters, highlighting the potential value of routinely available blood-based inflammatory indicators in ML-assisted PCa assessment (17). Although these studies support the feasibility of ML-based approaches in PCa, their endpoints mainly focused on disease detection or broad risk stratification rather than the preoperative prediction of LNM in patients with localized PCa. Therefore, there remains a need for clinically practical models specifically designed for the preoperative prediction of LNM using routinely available variables.

ML may provide a robust data-driven framework for integrating routinely available preoperative variables and modeling complex relationships among them (18, 19). Compared with conventional statistical approaches, ML algorithms may better capture nonlinear associations and interactions that are not readily identified using traditional models. For successful clinical translation, ML models should rely on data that are routinely available in clinical practice. Preoperative clinical characteristics, biopsy-derived pathological features, and standard laboratory parameters are routinely obtained before radical prostatectomy and impose no additional cost or burden on patients. These variables reflect not only tumor burden and histopathological aggressiveness but also systemic host responses, including inflammatory and nutritional status, which may be associated with metastatic potential. Accordingly, this study aimed to develop and externally validate ML-based models for the preoperative prediction of LNM in patients with localized PCa using routinely available clinical, biopsy, and laboratory data. By comparing multiple ML algorithms and incorporating model interpretability analyses, we aimed to identify an optimal predictive model to support individualized surgical planning, and ultimately enhance clinical decision-making in PCa management.

## 2 Methods

### 2.1 Study design and patients

We conducted a multicenter retrospective cohort study to develop and validate ML-based models for preoperative prediction of LNM in patients with localized PCa. This study was conducted and reported in accordance with the Transparent Reporting of a multivariable prediction model of Individual Prognosis Or Diagnosis-Artificial Intelligence (TRIPOD-AI) statement. Patients were consecutively recruited from two institutions. The primary cohort consisted of 632 consecutive patients who underwent RP with ePLND at the Affiliated Hospital of Qingdao University from March 2019 to December 2024. An independent external validation cohort comprised patients who underwent RP with ePLND at the Third Affiliated Hospital of Soochow University between July 2020 and September 2025 and met the same inclusion and exclusion criteria. Inclusion criteria comprised (1): histologically confirmed prostate adenocarcinoma diagnosed by systematic prostate biopsy (2); clinically localized disease prior to surgery; and (3) treatment with RP and ePLND. Exclusion criteria comprised (1): receipt of neoadjuvant therapy, including androgen deprivation therapy, chemotherapy, or radiotherapy, prior to radical prostatectomy (2); history of other malignant tumors; and (3) missing key preoperative variables, including clinical, biopsy, or laboratory data.

The reference standard for the presence or absence of LNM was exclusively based on postoperative histopathological examination of lymph nodes removed during ePLND. To ensure consistency in the outcome variable, ePLND was routinely performed at both the primary institution and the external validation center. The ePLND template encompassed the removal of lymph nodes from the internal iliac, external iliac, common iliac, and obturator regions. Patients in the primary cohort were divided into a training cohort (70%) and an internal validation cohort (30%) using a stratified random sampling approach. The external validation cohort was used exclusively to assess the generalizability of the developed models. This study was conducted in accordance with the Declaration of Helsinki and was approved by the Ethics Committees of the Affiliated Hospital of Qingdao University and the Third Affiliated Hospital of Soochow University.

### 2.2 Data collection

Preoperative clinical characteristics and biopsy records were obtained from electronic medical records. The collected preoperative data on the patients included: age, sex, body mass index (BMI), total prostate-specific antigen (TPSA), prostate volume, prostate-specific antigen density (PSAD), clinical tumor stage (cT), biopsy Gleason grade group (bGG), systematic biopsy positive rate, preoperative white blood cell count (WBC), neutrophil count, lymphocyte count, monocyte count, platelet count, red blood cell count (RBC), hemoglobin, fibrinogen, albumin, and prealbumin. In addition, several derived inflammatory indices were defined as follows: neutrophil-to-lymphocyte ratio (NLR), neutrophil count divided by lymphocyte count; platelet-to-lymphocyte ratio (PLR), platelet count divided by lymphocyte count; lymphocyte-to-monocyte ratio (LMR), lymphocyte count divided by monocyte count; systemic immune-inflammation index (SII), platelet count × neutrophil count/lymphocyte count; and neutrophil percentage-to-albumin ratio (NPAR), neutrophil percentage (%)/albumin (g/dL).

### 2.3 Data splitting and preprocessing

The 632 patients from the Affiliated Hospital of Qingdao University constituted the primary dataset and were partitioned into training (70%) and internal validation (30%) cohorts using a stratified random sampling strategy to maintain a balanced distribution of LNM status. To address the class imbalance caused by the low incidence of LNM (14.56%) in the primary cohort, the Synthetic Minority Over-sampling Technique (SMOTE) was applied exclusively to the training cohort after data splitting. SMOTE was used to generate synthetic samples for the minority class (LNM-positive patients), thereby creating a balanced dataset for model development. The internal validation and external validation cohorts were left unchanged and retained their original imbalanced distributions, allowing an unbiased assessment of model performance under real-world data conditions. Prior to model development, all continuous variables were normalized using Z-score transformation to minimize the impact of scale differences on model performance. Feature selection was subsequently conducted using the least absolute shrinkage and selection operator (LASSO) regression, in which non-informative variables were penalized and eliminated through coefficient shrinkage. In addition, LASSO effectively addressed multicollinearity by retaining a single representative feature from groups of highly correlated variables, as identified through Spearman correlation analysis.

### 2.4 Model development and validation

To construct preoperative prediction models for LNM in patients with localized PCa, five ML algorithms were implemented, including Light Gradient Boosting Machine (LightGBM), eXtreme Gradient Boosting (XGBoost), NaiveBayes, Support Vector Machine (SVM), and logistic regression (LR). Model training and optimization were conducted exclusively on the training cohort. Eight predictors were selected for model development. Based on the 64 LNM-positive events in the training cohort, the corresponding events-per-variable was 8. Hyperparameter optimization for each algorithm was performed using a grid search strategy combined with 5-fold cross-validation across a predefined parameter space. The optimal hyperparameter configuration was determined according to the one-standard-error rule based on the mean area under the receiver operating characteristic curve (AUC) and subsequently used to train the final ML models on the entire training cohort. The final optimal hyperparameter values for the ML models are detailed in **Supplementary Table 1**. Internal validation was conducted using the independent internal validation cohort from the primary institution to evaluate model performance within the same data source. External validation was performed by directly applying the trained models to an independent cohort from the Third Affiliated Hospital of Soochow University, without further model retraining, to evaluate robustness and generalizability across institutions.

### 2.5 Model performance evaluation

Model performance was comprehensively evaluated in terms of discrimination, calibration, and clinical usefulness. Discriminative performance was primarily assessed using the AUC with corresponding 95% confidence intervals. Additional performance metrics, including accuracy, sensitivity, and specificity, were reported at optimal cutoff values determined by the Youden index. Model calibration was evaluated using calibration curves and quantified by the Brier score. Clinical utility was assessed using decision curve analysis (DCA), which estimated the net benefit of the model across a range of threshold probabilities relative to treat-all and treat-none strategies. The final model was identified based on a comprehensive comparison of these evaluation metrics.

### 2.6 Comparison with Briganti and MSKCC nomograms

To further evaluate the clinical relevance of the developed model, the optimal LightGBM model was compared with the established Briganti and MSKCC nomograms in both the internal and external validation cohorts. The baseline predicted probabilities of LNM for these established tools were computed utilizing their respective published algorithms based on preoperative variables. Discriminative ability was evaluated using the AUC with 95% CI.

### 2.7 Model interpretation

Interpretability analysis was conducted on the best-performing model using SHapley Additive exPlanations (SHAP). Global feature contributions were examined using SHAP summary plots, in which variables were ranked according to the mean absolute SHAP values across the study population. These plots additionally illustrated the direction and magnitude of each feature’s influence on the predicted probability of LNM.

### 2.8 Web-based prediction tool deployment

To support model accessibility and exploratory evaluation, an interactive web-based risk assessment tool was implemented using the Shiny framework. Built upon the finalized and optimized ML model, this tool allows clinicians to dynamically estimate the probability of LNM in patients with localized PCa. The web-based tool was designed for ease of use and accessibility, requiring no specialized software installation, and can be accessed through a standard web browser. Although it may assist preoperative risk stratification and shared decision-making in settings similar to the study population, the current version of the calculator should be considered exploratory and is provided for research and educational purposes only. Given the retrospective nature of our cohorts, its routine clinical application should be deferred until robust prospective validation in broader, diverse populations confirms its utility.

### 2.9 Statistical analysis

Continuous variables that did not conform to a normal distribution were reported as medians with interquartile range (IQR), while categorical variables were presented as frequencies and percentages. Comparisons between groups were performed using the Mann-Whitney U test for continuous variables and the chi-square test for categorical variables, as appropriate. The predictive performance of the models was quantified using AUC, while pairwise comparisons of AUCs were performed using DeLong’s test in both the internal and external validation cohorts. All statistical analyses were performed using two-sided tests, and p < 0.05 was considered statistically significant. The entire analytical pipeline was executed using R version 4.4.2 with the tidymodels framework and related packages, including bonsai, discrim, mxjqkit, mxjqcls2, rmda, and cvms. A feature-removal sensitivity analysis was performed to assess the influence of PLR on the final model. After excluding PLR, a reduced seven-feature LightGBM model was rebuilt and compared with the original eight-feature model in the internal and external validation cohorts using accuracy, AUC with 95% CI, sensitivity, and specificity.

## 3 Results

### 3.1 Patient characteristics

**Figure 1:**
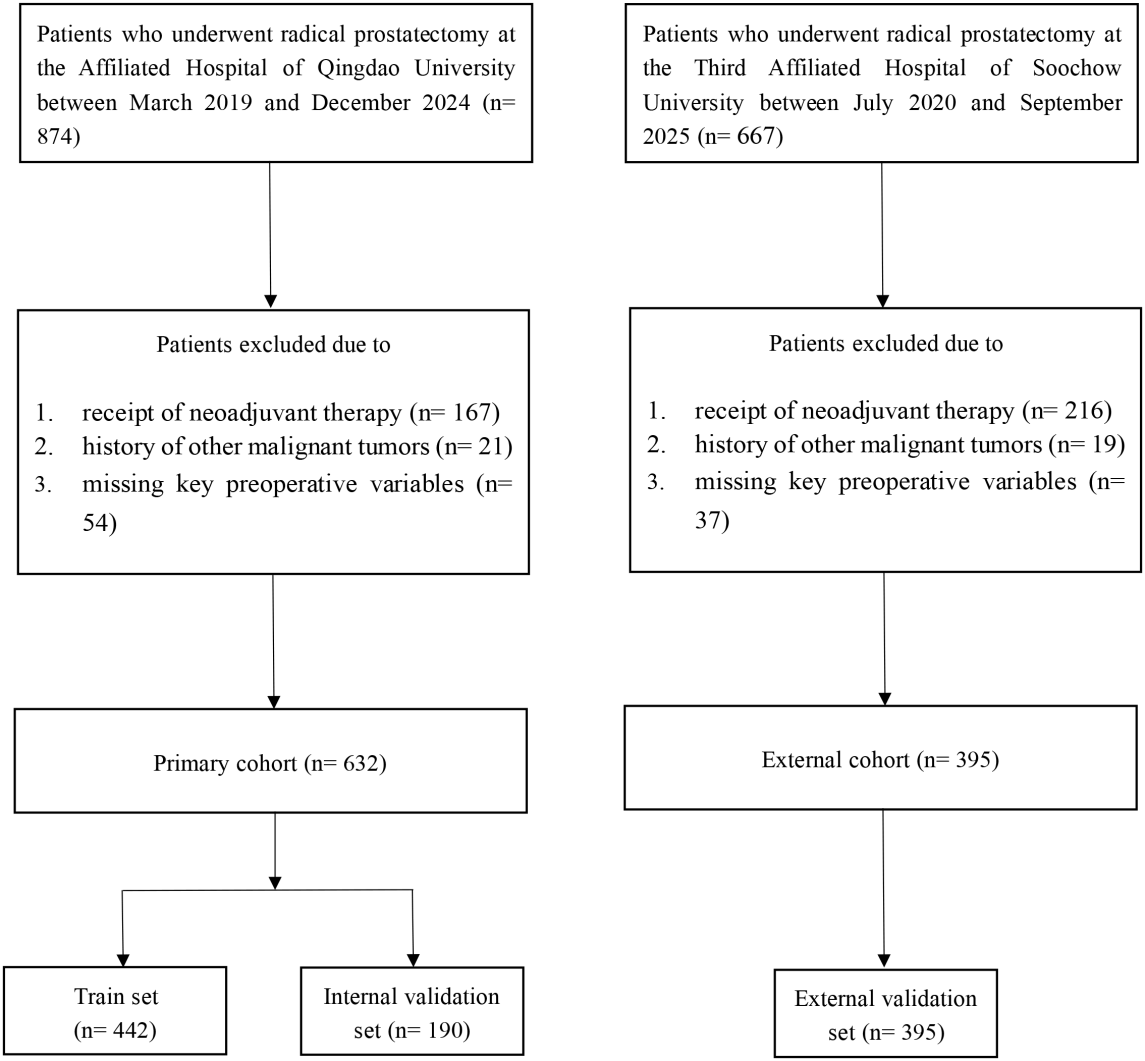
Flowchart showing the patient selection procedure.

The study utilized 632 patients from the Affiliated Hospital of Qingdao University as the primary cohort, with 92 patients diagnosed with LNM. The primary cohort was randomly divided into two subsets using the stratified random sampling approach: 70% of patients comprised the training cohort, while the remaining 30% constituted the internal validation cohort for constructing and screening the best predictive model. Additionally, an external cohort comprising 395 patients from the Third Affiliated Hospital of Soochow University was employed for external validation. Regarding the surgical nodal yield, the median number of lymph nodes removed and pathologically examined was 10 (IQR: 9-13) in the primary cohort, and 11 (IQR: 8-13) in the external validation cohort (p = 0.903). The robust nodal yield in both cohorts ensured the reliability of the pathologically confirmed LNM status as the reference standard. The flowchart illustrating patient selection is presented in **Figure 1**.

**Table 1** summarizes the baseline characteristics of patients in the primary cohort. Significant differences were identified for BMI, TPSA, PSAD, systematic biopsy positive rate, hemoglobin, lymphocyte count, NLR, PLR, SII, NPAR, bGG, and cT stage between patients with and without LNM. The baseline characteristics of the training, internal validation, and external validation cohorts are presented in **Table 2**. Notably, the p values shown in **Table 2** represent comparisons between the training and internal validation cohorts only. As presented in **Table 2**, the training and internal validation cohorts demonstrated comparable baseline characteristics. The stratified sampling approach ensured a well-balanced distribution of patients with LNM and without LNM across the two cohorts (14.5% vs. 14.7%, p = 0.933). In addition, no significant differences were identified across the remaining baseline variables, indicating that the two cohorts were well balanced and suitable for subsequent model development and internal validation.

**Table 1:** Baseline characteristics of the patients in the primary cohort.

Characteristics	LNM (–) (*n* = 540)	LNM (+) (*n* = 92)	P value
Age (years)	67 (65, 71)	66 (62, 73)	0.200
BMI (kg/m^2^)	25.34 (25.32, 25.34)	25.94 (23.77, 28.90)	0.002
TPSA (ng/ml)	14.22 (8.77, 28.06)	30.96 (15.88, 83.64)	< 0.001
Prostate volume (ml)	45.45 (34.35, 45.74)	45.45 (29.47, 49.75)	0.650
PSAD (ng/ml^2^)	0.36 (0.20, 0.81)	0.84 (0.34, 1.89)	< 0.001
Systematic biopsy positive rate	0.38 (0.14, 0.42)	0.42 (0.36, 0.71)	< 0.001
RBC (10^12^/L)	4.63 (4.32, 4.93)	4.58 (4.23, 4.92)	0.313
Hemoglobin (g/L)	143 (134, 151)	140 (130, 147)	0.022
WBC (10^9^/L)	6.31 (5.37, 7.40)	6.38 (5.51, 7.53)	0.388
Neutrophil (10^9^/L)	3.63 (2.90, 4.38)	3.94 (3.00, 4.97)	0.120
Lymphocyte (10^9^/L)	1.93 (1.53, 2.33)	1.76 (1.33, 2.18)	0.024
Monocyte (10^9^/L)	0.46 (0.37, 0.58)	0.46 (0.34, 0.57)	0.445
Platelet (10^9^/L)	212 (179, 252)	207 (174, 245)	0.690
NLR	1.90 (1.43, 2.54)	2.22 (1.51, 3.40)	0.024
PLR	110.40 (87.24, 141.39)	126.57 (93.67, 156.08)	0.011
LMR	4.04 (3.22, 5.21)	4 (3.05, 5.10)	0.230
SII	401.06 (283.35, 551.36)	450.41 (296.58, 770.70)	0.016
NPAR	14.03 (12.33, 15.67)	14.77 (12.67, 16.33)	0.032
Fibrinogen (g/L)	2.92 (2.56, 3.28)	3 (2.72, 3.35)	0.108
Albumin (g/L)	41.82 (38.90, 44.81)	41.88 (37.45, 44.33)	0.286
Prealbumin (g/L)	280.02 (248.88, 309.00)	275.85 (240.20, 304.62)	0.137
bGG, n (%)			< 0.001
1	113 (20.9%)	6 (6.5%)	
2	142 (26.3%)	4 (4.3%)	
3	108 (20%)	16 (17.4%)	
4	94 (17.4%)	24 (26.1%)	
5	83 (15.4%)	42 (45.7%)	
cT, n (%)			< 0.001
1	189 (35%)	9 (9.8%)	
2	297 (55%)	46 (50%)	
3	54 (10%)	37 (40.2%)	

LNM, lymph node metastasis; BMI, body mass index; TPSA, total prostate-specific antigen; PSAD, prostate-specific antigen density; RBC, red blood cell; WBC, white blood cell; NLR, neutrophil to lymphocyte ratio; PLR, platelet to lymphocyte ratio; LMR, Lymphocyte to monocyte ratio; SII, Systemic immune-inflammation; NPAR, neutrophil percentage-to-albumin ratio; bGG, biopsy Gleason grade group; cT clinical T stage.

**Table 2:** Demographic comparison of the training, internal validation, and external validation cohorts.

Characteristics	Train cohort (*n* = 442)	Internal validation cohort (*n* = 190)	P value	External validation cohort (n = 395)
Age (years)	67 (65, 72)	67 (64, 71)	0.655	72 (68, 76)
BMI (kg/m^2^)	25.34 (24.87, 25.85)	25.34 (25.34, 25.71)	0.958	23.74 (21.79, 25.72)
TPSA (ng/ml)	15.82 (9.27, 33.29)	15.80 (9.22, 34.92)	0.893	12.10 (7.72, 18.45)
Prostate volume (ml)	45.45 (34.55, 47.49)	45.45 (33.28, 45.45)	0.803	37.74 (27.48, 54.75)
PSAD (ng/ml^2^)	0.40 (0.22, 0.91)	0.37 (0.19, 0.93)	0.929	0.30 (0.19, 0.53)
Systematic biopsy positive rate	0.38 (0.20, 0.45)	0.38 (0.14, 0.43)	0.157	0.33 (0.17, 0.52)
RBC (10^12^/L)	4.62 (4.29, 4.92)	4.64 (4.35, 4.94)	0.241	4.63 (4.33, 4.92)
Hemoglobin (g/L)	142 (133, 150)	143 (134, 151)	0.457	143 (134, 150)
WBC (10^9^/L)	6.31 (5.4, 7.44)	6.36 (5.38, 7.44)	0.776	5.64 (4.77, 6.41)
Neutrophil (10^9^/L)	3.67 (2.92, 4.51)	3.59 (2.89, 4.41)	0.701	3.57 (2.99, 4.39)
Lymphocyte (10^9^/L)	1.92 (1.51, 2.29)	1.90 (1.53, 2.35)	0.628	1.41 (1.13, 1.75)
Monocyte (10^9^/L)	0.45 (0.37, 0.56)	0.48 (0.37, 0.60)	0.156	0.33 (0.27, 0.42)
Platelet (10^9^/L)	212 (178, 252)	208 (176, 251)	0.495	188 (156, 223)
NLR	1.96 (1.43, 2.60)	1.83 (1.42, 2.53)	0.538	2.56 (1.87, 3.51)
PLR	114.95 (87.12, 144.86)	105.83 (90.58, 140.22)	0.347	133.66 (104.34, 170.35)
LMR	4.05 (3.22, 5.21)	4.02 (3.11, 5.20)	0.589	4.2 (3.28, 5.34)
SII	412.50 (288.76, 577.15)	387.62 (281.11, 569.57)	0.404	484.51 (330.09, 680.46)
NPAR	14.11 (12.46, 15.89)	13.98 (12.10, 15.40)	0.148	15.55 (13.76, 16.94)
Fibrinogen (g/L)	2.95 (2.59, 3.32)	2.93 (2.58, 3.26)	0.678	2.70 (2.36, 3.10)
Albumin (g/L)	41.71 (38.79, 44.28)	42.01 (39.10, 45.40)	0.273	42.40 (40.10, 44.30)
Prealbumin (g/L)	279.90 (248.33, 306.15)	280.02 (246.73, 310.47)	0.213	279.70 (243.85, 308.80)
bGG, n (%)			0.340	
1	75 (17%)	44 (23.2%)		156 (39.5%)
2	104 (23.5%)	42 (22.1%)		68 (17.2%)
3	93 (21%)	31 (16.3%)		106 (26.8%)
4	81 (18.3%)	37 (19.5%)		52 (13.2%)
5	89 (20.1%)	36 (18.9%)		13 (3.3%)
cT, n (%)			0.143	
1	128 (29%)	70 (36.8%)		175 (44.3%)
2	249 (56.3%)	94 (49.5%)		177 (44.8%)
3	65 (14.7%)	26 (13.7%)		43 (10.9%)
label, n (%)			0.933	
LNM (-)	378 (85.5%)	162 (85.3%)		335 (84.8%)
LNM (+)	64 (14.5%)	28 (14.7%)		60 (15.2%)

BMI, body mass index; TPSA, total prostate-specific antigen; PSAD, prostate-specific antigen density; RBC, red blood cell; WBC, white blood cell; NLR, neutrophil to lymphocyte ratio; PLR, platelet to lymphocyte ratio; LMR, Lymphocyte to monocyte ratio; SII, Systemic immune-inflammation; NPAR, neutrophil percentage-to-albumin ratio; bGG, biopsy Gleason grade group; cT clinical T stage; LNM, lymph node metastasis. P values represent comparisons between the training and internal validation cohorts only; the external validation cohort is presented descriptively.

### 3.2 Feature selection for the predictive model

**Figure 2:**
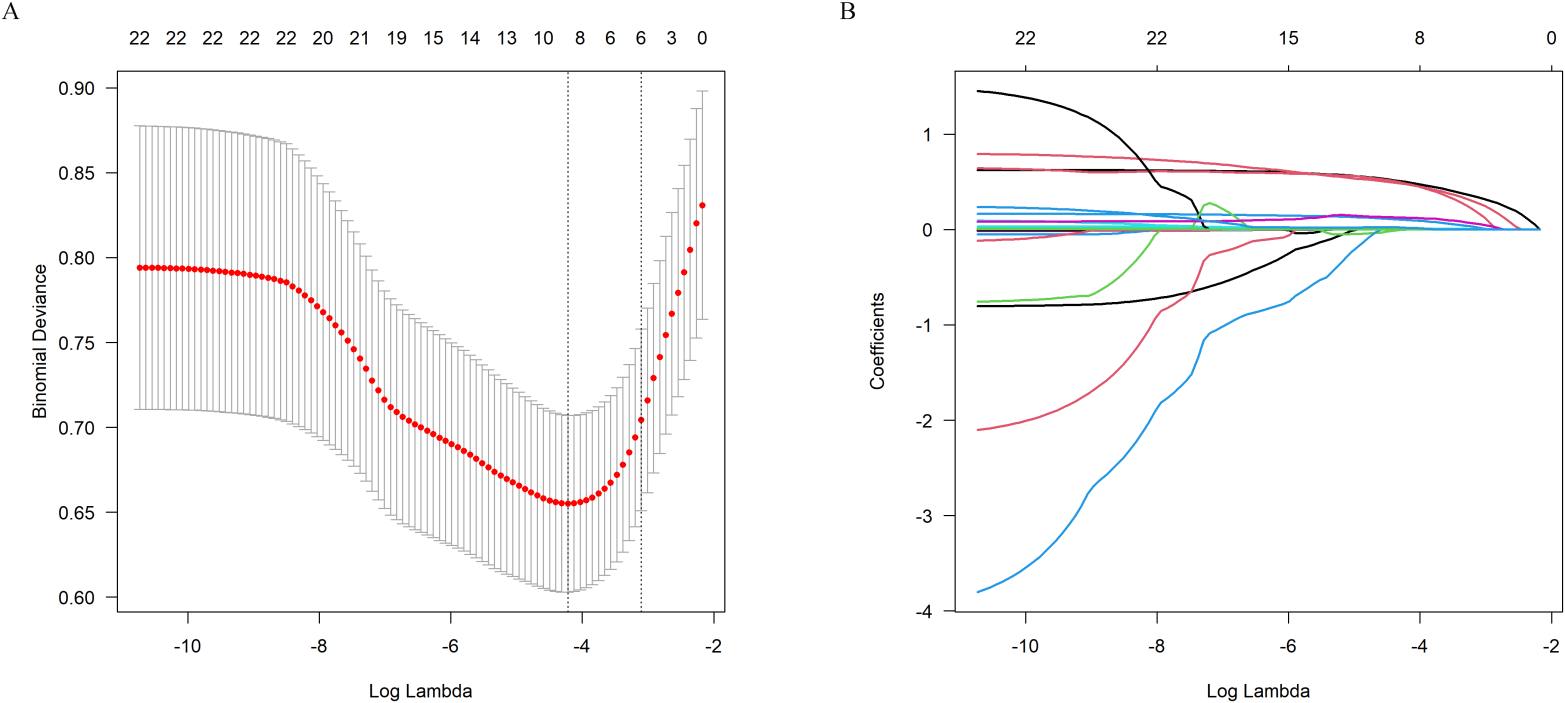
Feature selection using the least absolute shrinkage and selection operator (LASSO) regression. **(A)** Cross-validation plot for determining the optimal regularization parameter (Lambda) based on binomial deviance. **(B)** Coefficient paths demonstrating the shrinkage of predictor coefficients, with the number of non-zero coefficients decreasing as Lambda increases.

Feature selection was conducted using a combination of Spearman correlation analysis and the LASSO algorithm to identify the most informative variables. The optimal model was achieved at a lambda value of 0.0147, which yielded the best predictive performance. Through this selection process, the initial set of candidate variables was reduced to eight key predictors associated with LNM (**Figures 2A, B**). As summarized in **Table 3**, these eight variables were identified as potential predictors and were subsequently incorporated into the construction of the predictive models. Although PLR had the smallest non-zero coefficient among the selected predictors, feature-removal sensitivity analysis showed that excluding PLR decreased the AUC from 0.8613 to 0.8427 in the internal validation cohort and from 0.8148 to 0.7974 in the external validation cohort. These findings suggest that PLR provided modest additional discriminatory information and support its retention in the final eight-feature model (**Supplementary Table 2**).

**Table 3:** Feature selection results and coefficients for each feature.

Variable name	Coefficient
bGG	0.499942688
Systematic biopsy positive rate	0.483496061
cT	0.477749262
NLR	0.123815888
BMI	0.103170862
NPAR	0.020126515
TPSA	0.010007457
PLR	0.000350271

bGG, biopsy Gleason grade group; cT clinical T stage; NLR, neutrophil to lymphocyte ratio; BMI, body mass index; NPAR, neutrophil percentage-to-albumin ratio; TPSA, total prostate-specific antigen; PLR, platelet to lymphocyte ratio.

### 3.3 Model performance in the training and internal validation cohorts

**Figure 3:**
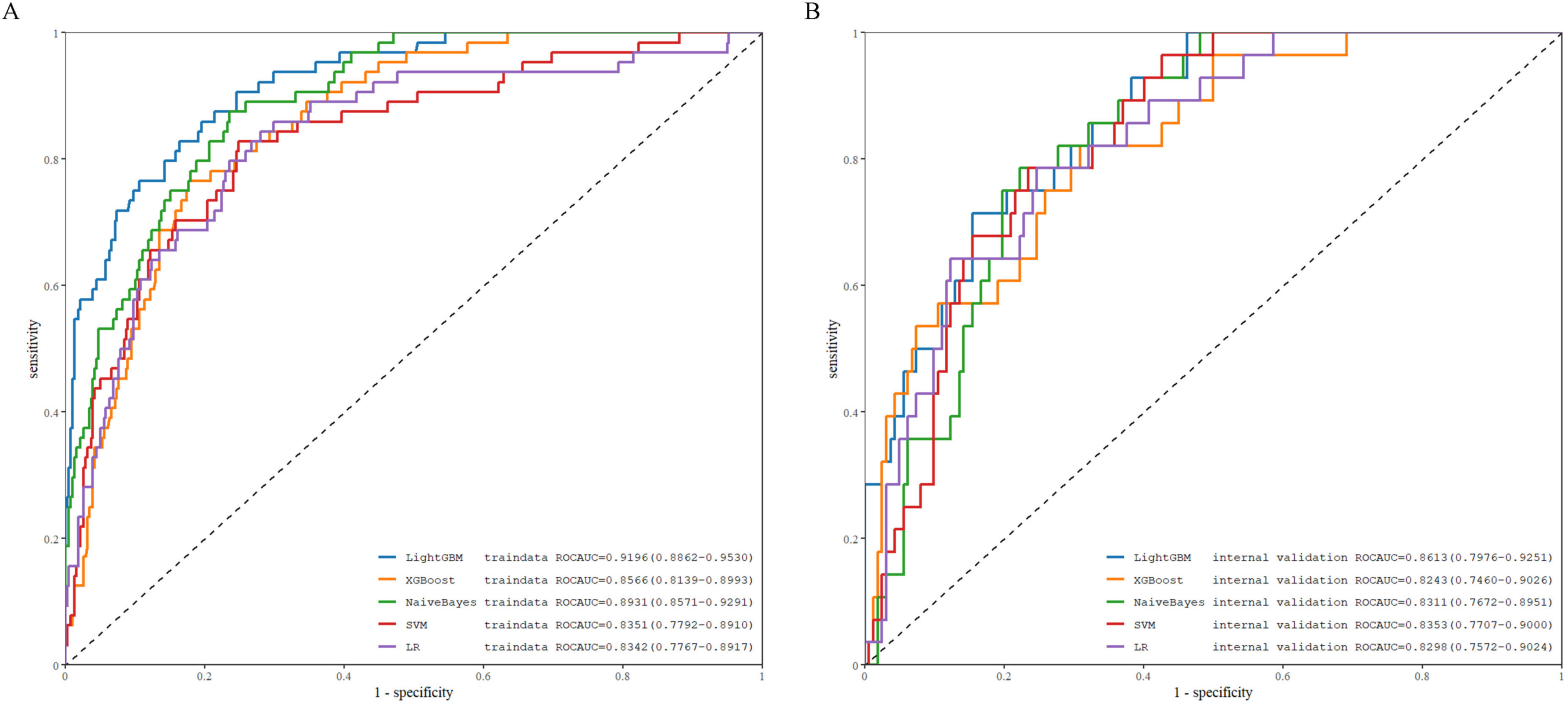
Receiver operating characteristic (ROC) curves of the five machine learning models in the training cohort **(A)** and internal validation cohort **(B)**. LightGBM, Light Gradient Boosting Machine; XGBoost, eXtreme gradient boosting; SVM, Support Vector Machine; LR, Logistic Regression.

Five ML models were constructed using the eight selected predictors as input variables. Model training was performed using the training cohort, followed by evaluation in the internal validation cohort to assess predictive performance. **Table 4** summarizes the AUC, accuracy, sensitivity, and specificity for each model in both the training cohort and internal validation cohort. Receiver operating characteristic (ROC) curves for the five models in the training and internal validation cohorts are presented in **Figures 3A, B**, respectively. In the training cohort, the LightGBM model demonstrated the strongest discriminative performance among all evaluated models, achieving an AUC of 0.9196 (95% CI: 0.8862–0.9530) with an accuracy of 0.8348. In the internal validation cohort, the LightGBM model continued to outperform the other models, maintaining stable performance with an AUC of 0.8613 (95% CI: 0.7976–0.9251) and an accuracy of 0.7842. Furthermore, DeLong’s test confirmed that the LightGBM model achieved a statistically significant improvement in AUC compared to the other four ML algorithms in the internal validation cohort (all p < 0.05; **Supplementary Table 3**).

**Table 4:** Comparison of the performance of machine learning models in the training, internal validation, and external validation cohorts.

Cohort	Model	Accuracy	AUC	95% CI	Sensitivity	Specificity
Training cohort						
	LightGBM	0.8348	0.9196	0.8862-0.9530	0.8281	0.8360
	XGBoost	0.8145	0.8566	0.8139-0.8993	0.7656	0.8228
	NaiveBayes	0.7805	0.8931	0.8571-0.9291	0.8750	0.7646
	SVM	0.7624	0.8351	0.7792-0.8910	0.8281	0.7513
	LR	0.7376	0.8342	0.7767-0.8917	0.8438	0.7196
Internal validation cohort						
	LightGBM	0.7842	0.8613	0.7976-0.9251	0.7143	0.7963
	XGBoost	0.7632	0.8243	0.7460-0.9026	0.6071	0.7901
	NaiveBayes	0.7316	0.8311	0.7672-0.8951	0.8214	0.7160
	SVM	0.7632	0.8353	0.7707-0.9000	0.7500	0.7654
	LR	0.7368	0.8298	0.7572-0.9024	0.7857	0.7284
External validation cohort						
	LightGBM	0.8506	0.8148	0.7477-0.8819	0.6333	0.8896
	XGBoost	0.8506	0.7721	0.7008-0.8433	0.5167	0.9104
	NaiveBayes	0.6380	0.7645	0.6933-0.8357	0.7500	0.6179
	SVM	0.7671	0.7559	0.6883-0.8236	0.5333	0.8090
	LR	0.8582	0.8084	0.7362-0.8807	0.7833	0.8716

AUC, area under the curve; 95% CI, 95% confidence intervals; LightGBM, light gradient boosting machine; XGBoost, eXtreme gradient boosting; SVM, Support Vector Machine; LR, Logistic Regression.

### 3.4 External validation performance

**Figure 4:**
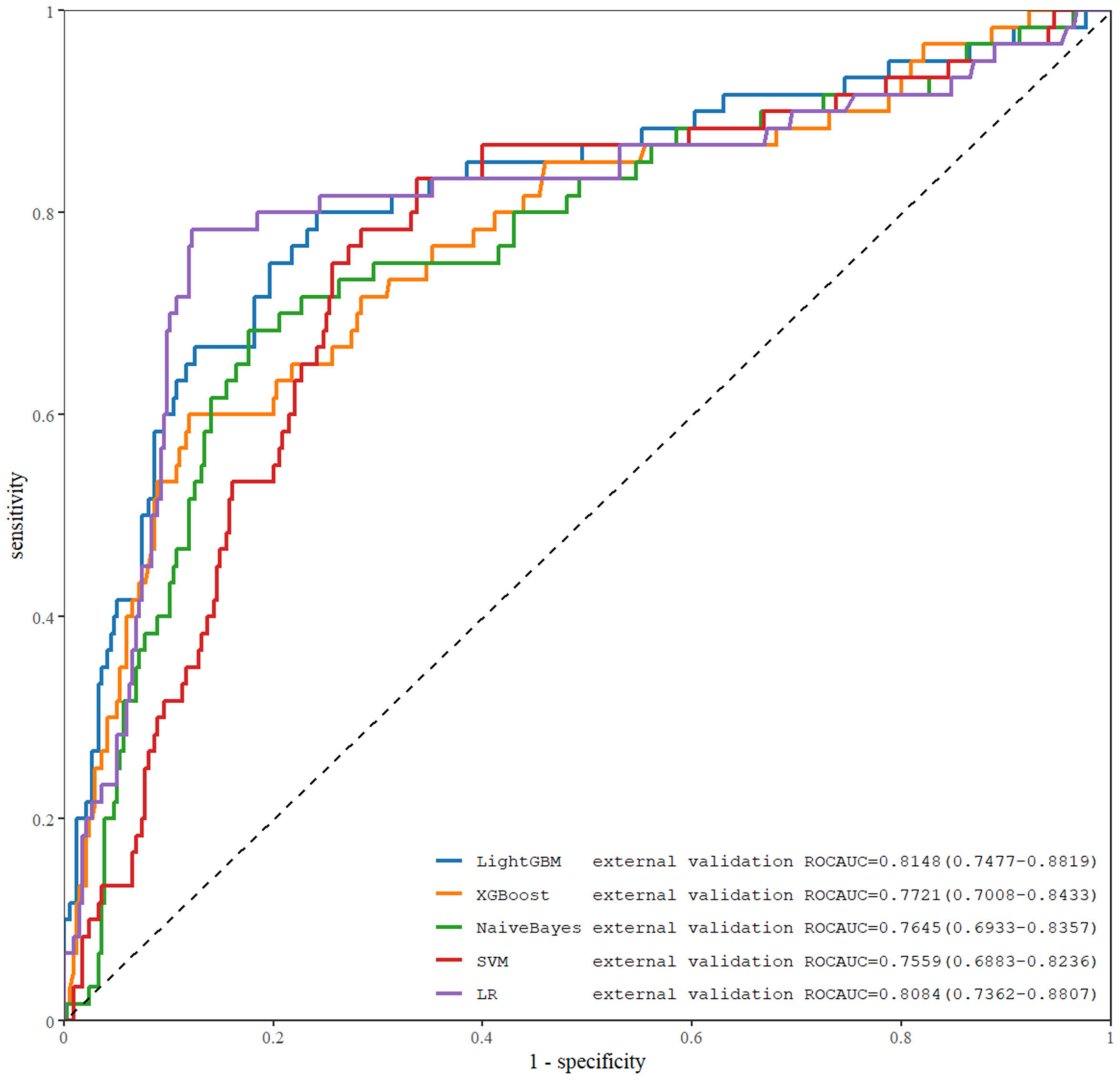
Receiver operating characteristic (ROC) curves of the five machine learning models in the external validation cohort. LightGBM, Light Gradient Boosting Machine; XGBoost, eXtreme gradient boosting; SVM, Support Vector Machine; LR, Logistic Regression.

The predictive performance of the five ML models in the external validation cohort is summarized in **Table 4**. Among the evaluated models, the LightGBM model achieved the highest AUC of 0.8148 (95% CI: 0.7477–0.8819) in the external validation cohort, while maintaining a favorable balance between sensitivity and specificity. ROC curves of the five models in the external validation cohort are presented in **Figure 4**. Pairwise comparisons using DeLong’s test in the external validation cohort showed that the LightGBM model significantly outperformed XGBoost, NaiveBayes, and SVM, while no significant difference was observed between LightGBM and LR (**Supplementary Table 4**).

### 3.5 Comparison with Briganti and MSKCC nomograms

We compared the discriminative performance of the LightGBM model with that of the established Briganti and MSKCC nomograms. As illustrated in **Supplementary Figures S1, S2**, the LightGBM model consistently demonstrated robust predictive performance across both validation cohorts. In the internal validation cohort (**Supplementary Figure S1**), LightGBM achieved an AUC of 0.8613 (95% CI: 0.7976-0.9251), compared to AUCs of 0.7740 (95% CI: 0.6666-0.8814) and 0.7888 (95% CI: 0.7020-0.8757) for the Briganti and MSKCC models, respectively. DeLong’s test showed that the LightGBM model had significantly better discriminative performance than both the Briganti and MSKCC nomograms in the internal validation cohort (both p < 0.05). In the independent external validation cohort (**Supplementary Figure S2**), the LightGBM model achieved the highest numerical AUC of 0.8148 (95% CI: 0.7477-0.8819), compared with 0.8033 (95% CI: 0.7397-0.8670) for the Briganti nomogram and 0.7973 (95% CI: 0.7348-0.8598) for the MSKCC nomogram. However, DeLong’s test indicated no statistically significant difference in discriminative performance between our model and the Briganti (p = 0.4045) or MSKCC (p = 0.2688) nomograms in the external validation cohort. These results suggest that the LightGBM model has predictive performance comparable to that of the established Briganti and MSKCC nomograms.

### 3.6 Calibration and clinical utility

**Figure 5:**
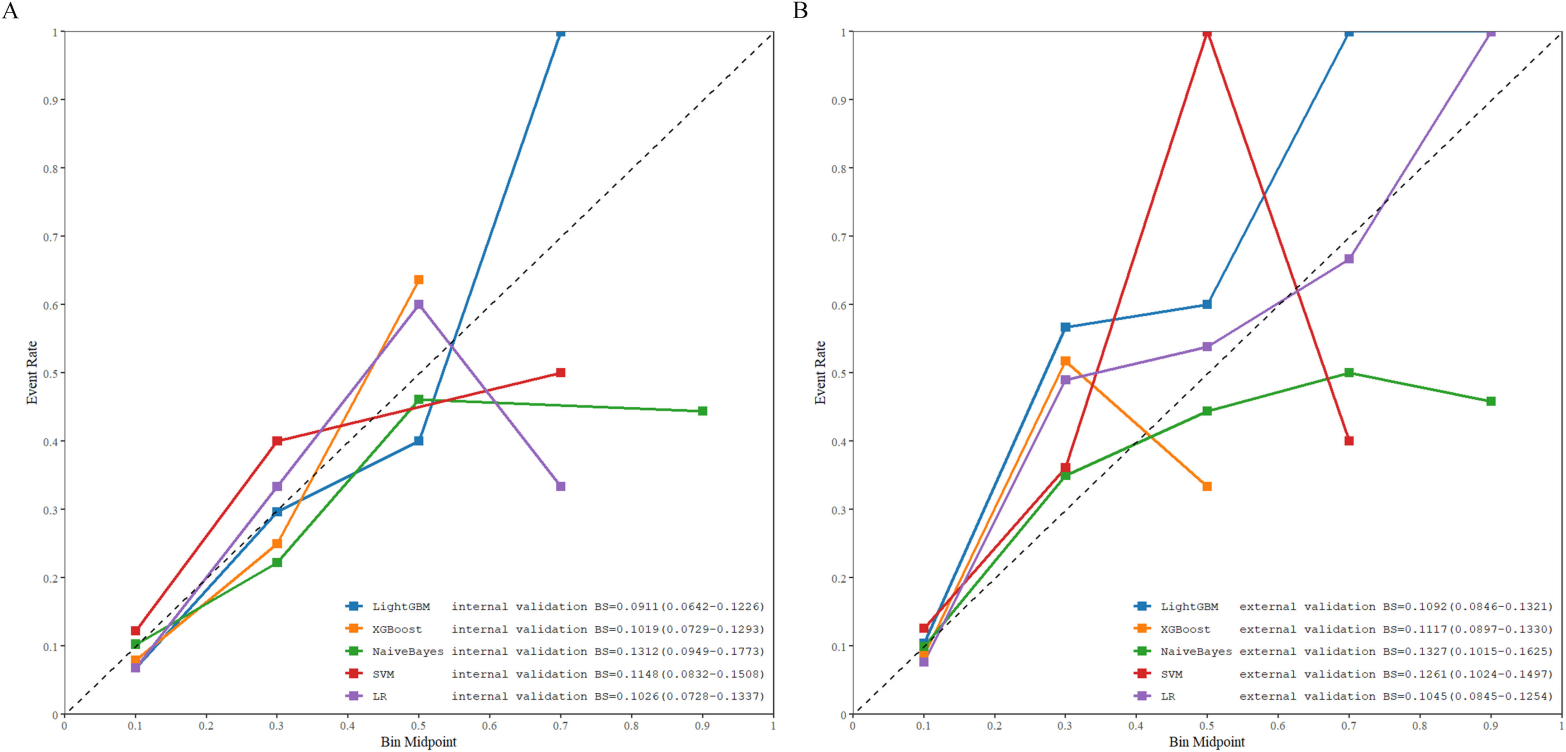
Calibration curves of the five machine learning models in the internal validation cohort **(A)** and external validation cohort **(B)**.

**Figure 6:**
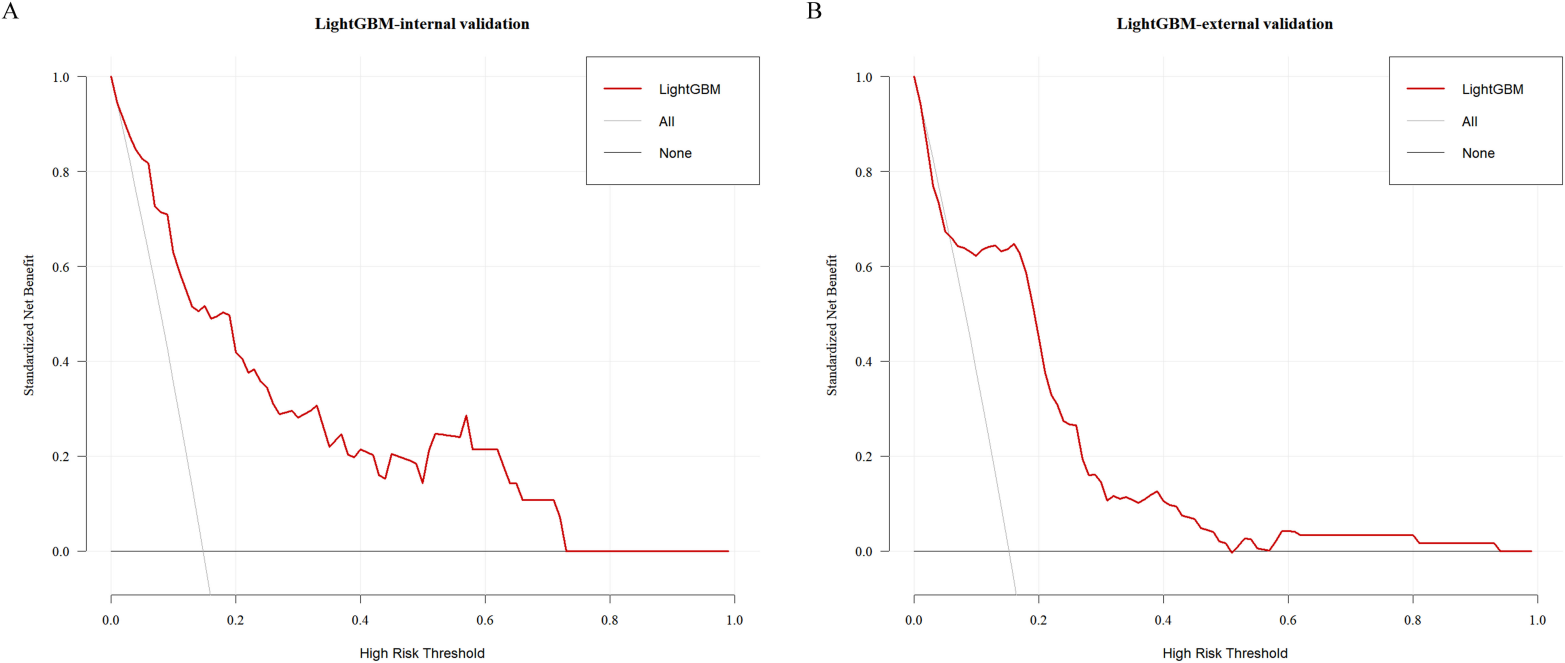
Decision curve analysis (DCA) of the LightGBM model in the internal validation cohort **(A)** and external validation cohort **(B)**.

Model calibration was evaluated in both the internal and external validation cohorts using calibration curves and the Brier scores (**Figures 5A, B**). In the internal validation cohort, the LightGBM model showed good calibration, with predicted probabilities closely matching the observed outcomes and a low Brier score of 0.0911. In the external validation cohort, the LightGBM model also maintained acceptable calibration, with a Brier score of 0.1092. Notably, LR achieved a slightly lower Brier score than LightGBM in the external validation cohort (0.1045 vs. 0.1092), suggesting marginally better calibration. Taken together, these findings indicate that LightGBM demonstrated acceptable calibration across validation cohorts, while LR showed a modest calibration advantage in the external validation cohort. The clinical utility of the selected LightGBM model was evaluated using DCA in both validation cohorts (**Figures 6A, B**). The model provided a higher standardized net benefit than the treat-all and treat-none strategies across a wide range of clinically relevant threshold probabilities in both the internal and external validation cohorts, suggesting its potential value for preoperative risk stratification and clinical decision-making.

### 3.7 Model interpretation using SHAP

**Figure 7:**
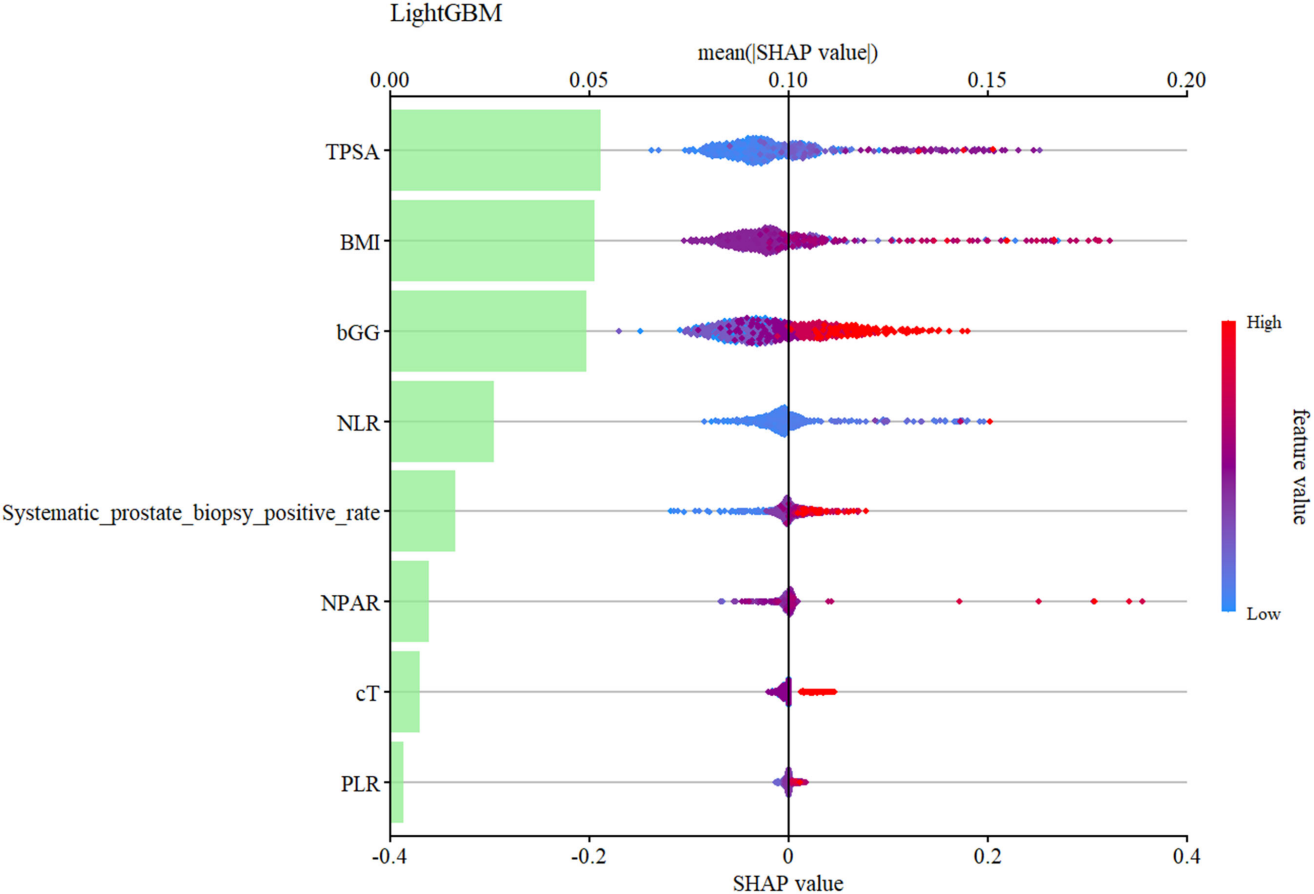
SHAP summary plot for the LightGBM model. The plot illustrates the relative importance and contribution direction for the LightGBM model. TPSA, total prostate-specific antigen; BMI, body mass index; bGG, biopsy Gleason grade group; NLR, neutrophil to lymphocyte ratio; NPAR, neutrophil percentage-to-albumin ratio; cT, clinical T stage; PLR, platelet to lymphocyte ratio.

To enhance model interpretability, SHAP was applied to the LightGBM model to quantify the contribution of individual features to the predicted risk of LNM in patients with localized PCa. The SHAP summary plot (**Figure 7**) illustrates both the relative importance of features, ranked by the mean absolute SHAP value, and the direction of their effects on model predictions. TPSA emerged as the most influential predictor, followed by BMI and bGG. Higher values of TPSA, BMI, and bGG were generally associated with increased predicted probabilities of LNM. In addition, inflammation-related biomarkers, including NLR and NPAR, as well as the systematic prostate biopsy positive rate, contributed meaningfully to the model’s predictions. cT stage and PLR also demonstrated measurable, albeit smaller, contributions to LNM risk estimation. Overall, SHAP analysis improved the interpretability of the LightGBM model by illustrating how tumor burden, pathological characteristics, and systemic inflammatory indicators contributed to the estimated risk of LNM.

### 3.8 Implementation of web calculator

**Figure 8:**
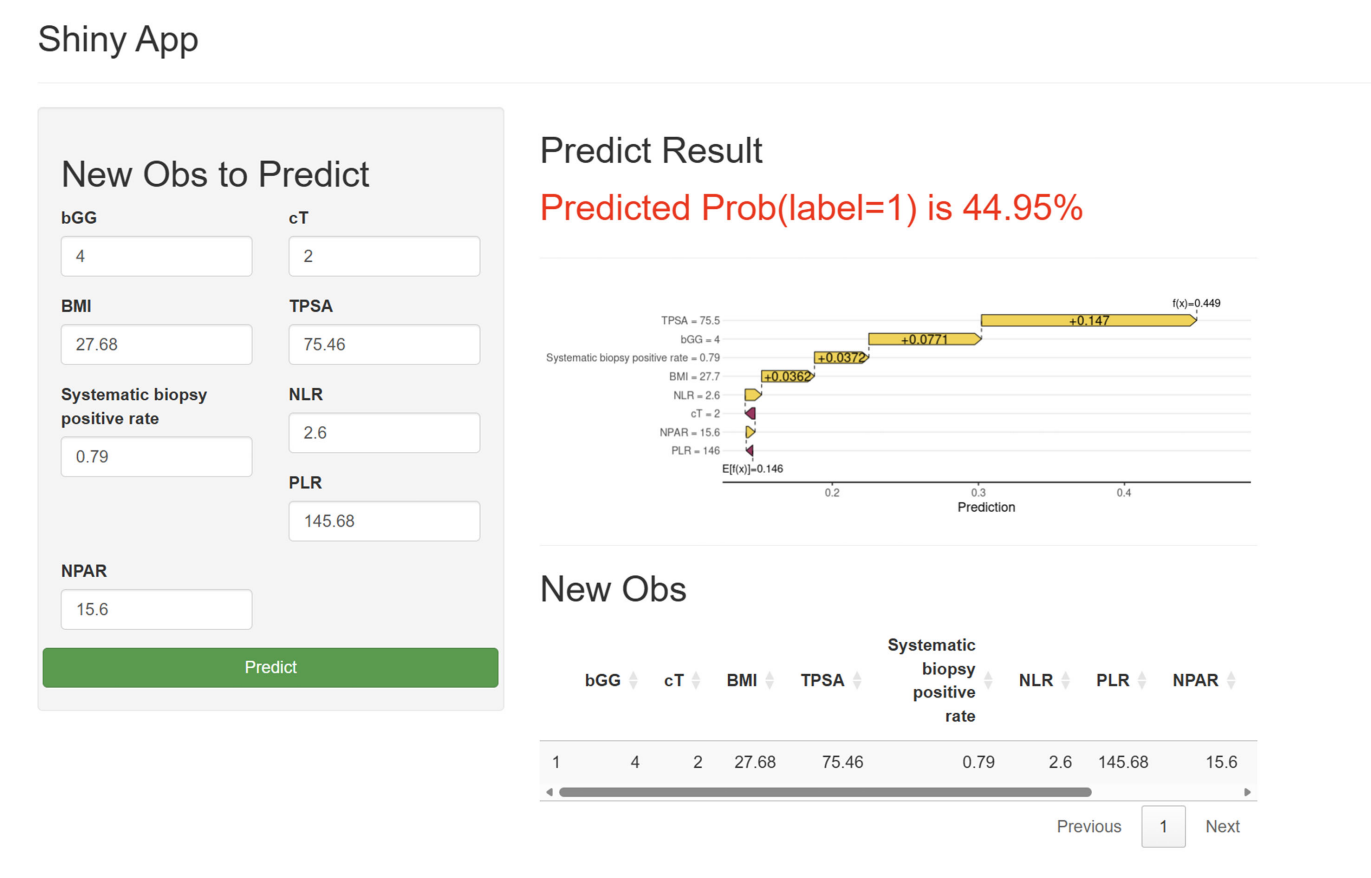
Web-based application for individualized prediction of LNM in localized PCa.

To improve the accessibility of the developed model, a web-based risk prediction tool was implemented based on the final LightGBM model. The online tool allows users to input individual preoperative clinical, biopsy, and laboratory variables and provides real-time estimation of the probability of LNM in patients with localized PCa (**Figure 8**). The web-based calculator is publicly accessible at: https://ml-model-1.shinyapps.io/online_model_520/.

## 4 Discussion

The presence of LNM in PCa is associated with substantially worse clinical outcomes, including disease progression, biochemical recurrence, cancer-specific mortality and changes in tumor staging and postoperative management (20–22). Consequently, accurate preoperative identification of lymph node metastasis is essential for appropriate risk stratification and for guiding decisions regarding pelvic lymph node dissection. In this multicenter retrospective study, we developed and externally validated interpretable ML models to preoperatively predict LNM in patients with localized PCa using routinely available clinical, biopsy, and laboratory data. Among the evaluated algorithms, the LightGBM model showed favorable and stable predictive performance, with robust discriminative ability, good calibration, and consistent clinical utility across both internal and external validation cohorts. Importantly, the incorporation of inflammation-related biomarkers and pathological features enabled the model to capture complex interactions beyond those addressed by traditional statistical approaches. To overcome the inherent “black-box” limitation of many ML models, SHAP was applied to enhance model interpretability. SHAP analysis further improved model transparency by illustrating the relative contribution and direction of individual predictors.

Several predictive tools and nomograms have been developed to estimate the risk of LNM in patients with PCa, among which the Briganti and MSKCC nomograms are the most widely adopted in clinical practice (23, 24). These models are primarily derived from large surgical cohorts and incorporate established clinicopathological variables such as PSA levels, cT stage, biopsy Gleason score, and the extent of positive biopsy cores (10). In the present study, the LightGBM model achieved a significantly higher AUC than both the Briganti and MSKCC nomograms in the internal validation cohort, suggesting improved discrimination within the primary study population. However, this advantage was attenuated in the independent external validation cohort, where LightGBM retained the highest numerical AUC but showed no statistically significant difference compared with the Briganti or MSKCC nomograms. Similarly, among the evaluated ML models, LR also demonstrated strong external validation performance, with slightly higher accuracy, sensitivity, and calibration performance than LightGBM, although the AUC difference between LR and LightGBM was not statistically significant. Therefore, our findings should not be interpreted as evidence that LightGBM was uniformly superior to simpler or established models across all metrics and validation settings. Notably, the external validation cohort differed from the training cohort in several baseline characteristics, including age, BMI, TPSA, prostate volume, and inflammation-related biomarkers such as NLR, PLR, and NPAR. These differences may reflect variations in patient populations and institutional clinical practice between the two participating centers. Despite this inter-cohort heterogeneity, the LightGBM model maintained favorable discriminative performance in the external validation cohort, suggesting acceptable robustness and potential generalizability across different clinical settings. The attenuation of the performance advantage observed in the external validation cohort may therefore be attributable, at least in part, to differences in patient characteristics between institutions rather than instability of the model itself. Nevertheless, LightGBM was selected as the primary model for subsequent SHAP-based interpretation and web-based calculator development because of its favorable internal validation performance, highest numerical external AUC, acceptable calibration, and compatibility with TreeSHAP-based interpretation of nonlinear relationships and potential feature interactions. These findings also highlight the importance of balanced model evaluation while recognizing the potential limitations of conventional nomograms. First, their reliance on linear modeling assumptions may restrict the ability to capture complex nonlinear relationships and higher-order interactions among predictors, which are common in heterogeneous clinical datasets (25, 26). Second, most existing nomograms focus predominantly on tumor-related variables and do not incorporate systemic host-related factors, such as inflammatory or nutritional biomarkers, which have been increasingly implicated in tumor aggressiveness and metastatic potential (27, 28).

The methodological characteristics of LightGBM may partly explain its favorable performance in this study. First, LightGBM is a tree-based gradient boosting algorithm capable of modeling complex nonlinear relationships and high-order interactions among predictors without requiring explicit specification (29). Second, LightGBM incorporates advanced techniques such as histogram-based feature binning and leaf-wise tree growth, which improve computational efficiency and enable more effective learning from structured clinical data (30). These features allow the model to handle correlated predictors and mixed data types more robustly than conventional regression-based approaches, and may have facilitated the integration of tumor-related factors with inflammation-related and nutrition-related biomarkers in the present study. Beyond predictive performance, interpretability is essential for the clinical acceptance of ML models. Therefore, SHAP analysis was used to quantify the relative contribution and direction of each predictor in the LightGBM model. Importantly, SHAP values should be interpreted as model-based explanations rather than evidence of causal relationships or biological mechanisms. Accordingly, the SHAP analysis in this study primarily clarified how the model used available preoperative variables to generate individualized LNM risk estimates.

In the SHAP analysis, TPSA showed the greatest contribution to the LightGBM model output among the included predictors. Elevated TPSA levels reflect increased tumor burden and biological aggressiveness and have been consistently associated with adverse pathological features in PCa, including extracapsular extension and lymph node involvement (31, 32). Similarly, bGG and cT were identified as key contributors, underscoring the critical role of tumor differentiation and local tumor extent in determining metastatic potential. Higher bGG reflects poorer tumor differentiation and more aggressive biological behavior, which has been consistently associated with increased risks of lymph node involvement and disease progression in PCa (33). cT stage, representing the extent of local tumor invasion, further captures the anatomical progression of the disease and has been widely recognized as an important predictor of nodal metastasis (34, 35). The prominent contribution of these established clinicopathological variables supports the clinical face validity of the model.

Notably, our LightGBM model identified several inflammation-related biomarkers, including NLR, PLR, and NPAR, as important contributors to predictive performance. This observation is broadly consistent with previous studies reporting associations between these markers and adverse oncologic outcomes in PCa and other malignancies (36, 37). For instance, elevated NLR and PLR have been associated with poorer oncologic outcomes in PCa, possibly by reflecting an imbalanced host immune response that may facilitate tumor growth and metastasis (38, 39). Similarly, NPAR, which combines inflammatory and nutritional information, has been correlated with adverse outcomes in other malignancies (37, 40). While our model does not establish a causal relationship, the predictive utility of these markers suggests that the systemic host environment they represent contains valuable information for estimating LNM risk. Their inclusion allows the model to capture a dimension of patient status beyond tumor-specific characteristics.

In addition, BMI emerged as an important predictor in our model, as highlighted by the SHAP analysis. Growing evidence suggests that obesity and metabolic dysregulation are associated with more aggressive tumor behavior and poorer oncologic outcomes in PCa (41, 42). However, BMI is a crude anthropometric measure and does not directly characterize the biological pathways potentially linking metabolic status with tumor dissemination. In our model, BMI should therefore be viewed primarily as a clinically accessible predictor that may summarize broader host-related information, rather than as a mechanistic indicator of LNM. The systematic prostate biopsy positive rate also contributed to LNM risk prediction, likely reflecting tumor volume and the spatial extent of cancer involvement within the prostate. A higher proportion of positive biopsy cores has been repeatedly associated with advanced pathological stage and nodal involvement, supporting its inclusion as a clinically intuitive predictor (43).

From a clinical perspective, accurate preoperative prediction of LNM is essential for optimizing surgical planning and improving individualized management in patients with localized PCa. By integrating routinely available clinical, biopsy, and laboratory variables, the proposed LightGBM-based model provides a practical and individualized approach to preoperative risk stratification. For patients identified as high risk by the model, clinicians may consider more meticulous preoperative evaluation and an appropriately extended lymph node dissection, whereas patients classified as low risk may potentially avoid unnecessary surgical intervention. To improve accessibility and support exploratory evaluation, we further developed a publicly accessible web-based risk calculator based on the LightGBM model. This tool enables clinicians to input individual patient data and obtain real-time estimates of LNM risk through a user-friendly interface without the need for specialized software. The web-based calculator is intended to serve as an exploratory decision-support tool to assist clinicians in risk communication and shared decision-making in settings similar to the study population, rather than to replace clinical judgment.

Several limitations of our study should be noted. First, the retrospective design and reliance on electronic medical records may have introduced selection and information biases despite the use of consecutive patient inclusion and clearly defined eligibility criteria. In addition, the study population was derived from two Chinese centers, which may limit the generalizability of the model to broader populations. Second, the relatively low number of LNM events may have increased the risk of overfitting, although several methodological strategies were used to mitigate this risk. Larger prospective cohorts with more LNM events are needed to further validate the stability and generalizability of the model. Third, although ePLND template was intended across both institutions, confirming exact anatomical boundaries retrospectively is challenging. Inter-center variations in surgical techniques and pathological specimen handling may have caused fluctuations in nodal yield, potentially introducing ascertainment bias regarding the true LNM status. Fourth, due to data availability constraints, certain potentially informative preoperative variables, including multiparametric MRI features, PI-RADS scores, PSMA PET findings, and molecular or genomic biomarkers, were not incorporated. Although SHAP was used to improve model interpretability, the identified associations should not be interpreted as causal relationships. Therefore, prospective multicenter studies with larger and more diverse populations are needed to confirm the clinical utility of the proposed model and to evaluate its impact on surgical decision-making and patient outcomes.

## 5 Conclusions

This study developed and externally validated an interpretable LightGBM model for the preoperative prediction of LNM in localized PCa. The model showed competitive performance and achieved discrimination comparable to that of the Briganti and MSKCC nomograms. Although these findings support its potential value for preoperative risk stratification in similar surgical settings, the generalizability of the model remains limited by the retrospective design and the inclusion of only Chinese high-volume surgical cohorts. Therefore, the web-based calculator should currently be regarded as an exploratory tool, and prospective validation in larger and more diverse populations is required before routine clinical implementation.

## Data Availability

The raw data supporting the conclusions of this article will be made available by the authors, without undue reservation.
