# Supplementary material for: Using interpretable machine learning model to predict lymph node metastasis in patients with localized prostate cancer
Source: Front Oncol. 2026 Jul 22;16:1804019. doi: 10.3389/fonc.2026.1804019 (PMC13437460; doi:10.3389/fonc.2026.1804019)
Supplement: Supplementary file 1 [file DataSheet1.docx]

Supplementary Material

**Supplementary Table S1.** Optimal Hyperparameters values for the machine learning models.

| **Model** | **Hyperparameter** | **Optimal Value** |
| --- | --- | --- |
| LightGBM | mtry | 8 |
|  | trees | 486 |
|  | min_n | 10 |
|  | tree_depth | 5 |
|  | learn_rate | 0.064785 |
|  | loss_reduction | 0.88808 |
| XGBoost | mtry | 6 |
|  | min_n | 19 |
|  | tree_depth | 5 |
|  | learn_rate | 0.088528 |
|  | loss_reduction | 0.81828 |
|  | sample_size | 0.89656 |
| NaiveBayes | smoothness | 1.8804 |
|  | Laplace | 4.8911 |
| SVM | cost | 20.688 |
|  | rbf_sigma | 0.086036 |

LightGBM, light gradient boosting machine; XGBoost, eXtreme gradient boosting; SVM, Support Vector Machine.

**Supplementary Table S2.** Sensitivity analysis comparing the performance of the LightGBM model with and without PLR.

| **Cohort** | **Model** | **Accuracy** | **AUC** | **95% CI** | **Sensitivity** | **Specificity** |
| --- | --- | --- | --- | --- | --- | --- |
| Internal validation cohort | | |  |  |  |  |
|  | LightGBM with PLR | 0.7842 | 0.8613 | 0.7976-0.9251 | 0.7143 | 0.7963 |
|  | LightGBM without PLR | 0.8052 | 0.8427 | 0.7645-0.9210 | 0.7857 | 0.8086 |
| External validation cohort | | |  |  |  |  |
|  | LightGBM with PLR | 0.8506 | 0.8148 | 0.7477-0.8819 | 0.6333 | 0.8896 |
|  | LightGBM without PLR | 0.8405 | 0.7974 | 0.7252-0.8697 | 0.4667 | 0.9075 |

PLR, platelet-to-lymphocyte ratio; AUC, area under the receiver operating characteristic curve; CI, confidence interval.

**Supplementary Table S3.** Pairwise comparison of AUCs among machine learning models using DeLong’s test in the internal validation set.

| Model | XGBoost | NaiveBayes | SVM | LR |
| --- | --- | --- | --- | --- |
| LightGBM | 0.0230 | 0.0280 | 0.0187 | 0.0239 |
| XGBoost |  | 0.1846 | 0.1763 | 0.1867 |
| NaiveBayes |  |  | 0.1894 | 0.1835 |
| SVM |  |  |  | 0.1852 |

LightGBM, light gradient boosting machine; XGBoost, eXtreme gradient boosting; SVM, Support Vector Machine; LR, Logistic Regression**.**

**Supplementary Table S4.** Pairwise comparison of AUCs among machine learning models using DeLong’s test in the external validation set.

| Model | XGBoost | NaiveBayes | SVM | LR |
| --- | --- | --- | --- | --- |
| LightGBM | 0.0262 | 0.0219 | 0.0052 | 0.7510 |
| XGBoost |  | 0.8040 | 0.5896 | 0.1206 |
| NaiveBayes |  |  | 0.7192 | 0.0627 |
| SVM |  |  |  | 0.0121 |

LightGBM, light gradient boosting machine; XGBoost, eXtreme gradient boosting; SVM, Support Vector Machine; LR, Logistic Regression**.**

**Supplementary Figure S1.** Receiver operating characteristic (ROC) curves comparing the predictive performance of the LightGBM model, Briganti model, and MSKCC model in the internal validation cohort.

**Supplementary Figure S2.** Receiver operating characteristic (ROC) curves comparing the predictive performance of the LightGBM model, Briganti model, and MSKCC model in the external validation cohort.
